# Development of the aganglionic colon following surgical rescue in a cell therapy model of Hirschsprung disease in rat

**DOI:** 10.1242/dmm.050055

**Published:** 2023-04-27

**Authors:** John B. Furness, Enie Lei, Billie Hunne, Cameron D. Adams, Alan J. Burns, Jill Wykosky, Therese E. Fazio Coles, Linda J. Fothergill, Juan C. Molero, Ruslan V. Pustovit, Lincon A. Stamp

**Affiliations:** ^1^ Florey Institute of Neuroscience and Mental Health, Parkville, Victoria 3010, Australia; ^2^ Department of Anatomy & Physiology, University of Melbourne, Parkville, Victoria 3010, Australia; ^3^ Gastroenterology Drug Discovery Unit, Takeda Pharmaceutical Company International Inc., Boston, MA 02138, USA

**Keywords:** Hirschsprung disease, Stem cell therapy, Colon, Intestinal bypass, Enteric neurons, Enteric nervous system

## Abstract

Patients with Hirschsprung disease lack enteric ganglia in the distal colon and propulsion of colorectal content is substantially impaired. Proposed stem cell therapies to replace neurons require surgical bypass of the aganglionic bowel during re-colonization, but there is inadequate knowledge of the consequences of bypass. We performed bypass surgery in *Ednrb*^−/−^ Hirschsprung rat pups. Surgically rescued rats failed to thrive, an outcome reversed by supplying electrolyte- and glucose-enriched drinking water. Histologically, the bypassed colon had normal structure, but grew substantially less in diameter than the functional region proximal to the bypass. Extrinsic sympathetic and spinal afferent neurons projected to their normal targets, including arteries and the circular muscle, in aganglionic regions. However, although axons of intrinsic excitatory and inhibitory neurons grew into the aganglionic region, their normally dense innervation of circular muscle was not restored. Large nerve trunks that contained tyrosine hydroxylase (TH)-, calcitonin gene-related peptide (CGRP, encoded by *Calca* or *Calcb*)-, neuronal nitric oxide synthase (nNOS or NOS1)-, vasoactive intestinal peptide (VIP)- and tachykinin (encoded by *Tac1*)-immunoreactive axons occurred in the distal aganglionic region. We conclude that the rescued *Ednrb*^−/−^ rat provides a good model for the development of cell therapies for the treatment of Hirschsprung disease.

## Teaser

**Summary:** Surgical rescue of *Ednrb*^−/−^ rats, a model for Hirschsprung disease, required dietary intervention for survival and showed reduced colon diameter in the aganglionic region, overall normal histology and changes in innervation.

## INTRODUCTION

In Hirschsprung disease, there is failure of the enteric nervous system of the distal bowel to form during development. This results in a variable length of the colon and rectum in which there are no enteric ganglia and, thus, an absence of the propulsion of content that is normally regulated through the enteric nervous system. An accumulation of feces occurs in the bowel proximal to the aganglionosis. The symptoms of Hirschsprung disease include abdominal distension, constipation, vomiting and retarded growth. The disease is treated surgically by removal of the aganglionic part of the bowel (Swenson et al., 1949; Duhamel, 1960; Soave, 1964; Swenson, 2002). If the condition is untreated, many children die, generally from septicemia that is a result of severe enterocolitis or perforation of the bowel. Although surgical removal or bypass of the aganglionic region of bowel is life-saving, a high proportion of patients have ongoing complications, including constipation, fecal incontinence, bladder dysfunction and Hirschsprung disease-associated enterocolitis (Skinner, 1996; Gosain and Brinkman, 2015), that impair their quality of life. The complications possibly arise from the early-life surgery to anastomose the ganglionic bowel with the final segment of the anal canal after the removal of the aganglionic region, because the surgery inevitably interferes with the normal anatomical relationships of the distal rectum, including connections of pelvic nerves and pelvic ganglia. The pelvic nerves have important roles in the control of colorectal function (Callaghan et al., 2018).

It has been speculated that a cell therapy that restores the enteric nervous system and permits retention of the aganglionic bowel and its normal anatomical relations may provide a better outcome (Heanue and Pachnis, 2007; Burns and Thapar, 2014; Fattahi et al., 2016; Mueller and Goldstein, 2022; Stamp et al., 2022). To apply cell therapy in human infants, it is anticipated that the aganglionic colon will need to be bypassed for weeks or months to allow time for implanted neural precursor cells to repopulate the aganglionic colon, to form nerve circuits that integrate with extrinsic nerve connections and to restore function. With this in mind, we have developed a bypass surgery in Hirschsprung rats that extends the lifetimes of the animals from the 3-5 weeks of survival without surgery to 6 months or more (Stamp et al., 2015, 2022). Currently, we do not know the fate of the aganglionic region of colon that has been bypassed, in particular, whether it maintains its integrity and whether it becomes innervated by endogenous neurons, including from the more proximal ganglionated region, the pelvic nerves and sympathetic pathways. Thus, there is lack of knowledge of the substrate into which stem cells would be implanted to restore colorectal function.

In the present work, we have investigated the colons of *Ednrb*^−/−^ rats (Ceccherini et al., 1995; Gariepy et al., 1996), which mimic the *EDNRB^−/−^* phenotype in human (Lamont et al., 1989; Kusafuka and Puri, 1997; Amiel et al., 2008), in that variable lengths of the distal colon lack enteric neurons, the aganglionic region is non-propulsive, the more proximal bowel is distended and death follows in weeks (in rat) or months (in human) if surgical bypass of the aganglionic region is not performed.

## RESULTS

### Inheritance, colon appearance and animal growth

The inheritance of the *Ednrb*^−/−^ knockout (KO) condition with Het×Het breeding was 22.6% (72 of 319 live born animals from 28 litters), close to that predicted by Mendelian inheritance, determined by genotyping progeny, with a male:female ratio of 39:33 (1.18:1). There were 75/319 wild-type (WT) animals and 172/319 heterozygotes. Investigation of unoperated KO rats at 3-4 weeks of age showed obvious pathology (Fig. S2A). In 70% of KO rats, there was a narrow distal colon and rectum, and proximal to this region, there was dilatation of the colon and an enlarged cecum. It was common that the distal ileum was also enlarged (Fig. S2A,B). In 20% of cases, enlargement extended almost the full length of the large intestine (Fig. S2C).

**Fig. 1.**
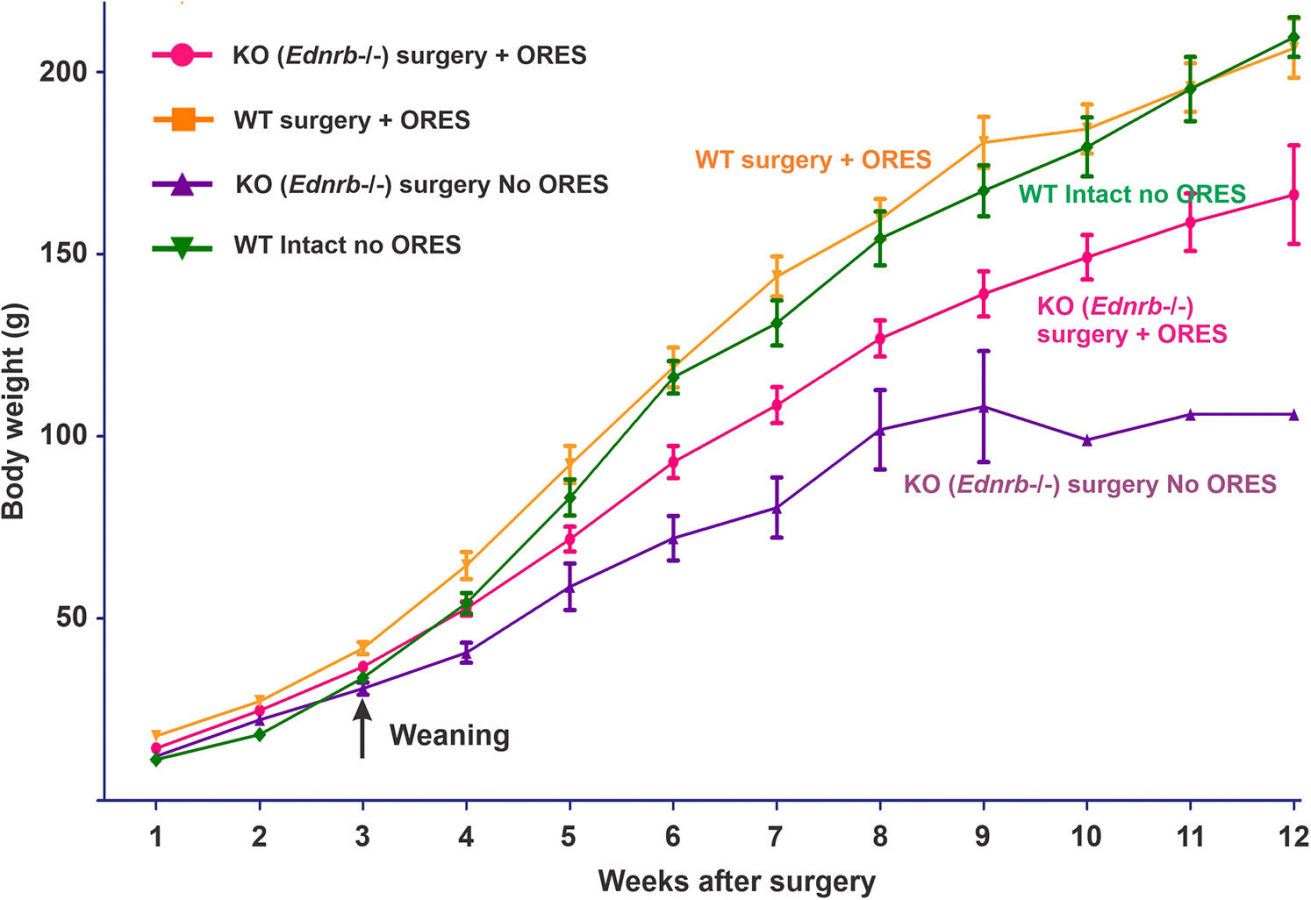
**Growth curves of *Ednrb*^−/−^ and WT rats from the *Ednrb* colony, showing the effect of supplementation with an oral rehydration and energy solution.** *Ednrb*^−/−^ (KO) Hirschsprung rats [no oral rehydration and energy solution (ORES) supplementation] failed to thrive after rescue surgery and did not gain weight after 8 weeks of age (*n*=10). *Ednrb*^−/−^ Hirschsprung rats subjected to rescue surgery that consumed ORES grew steadily and at 11 weeks of age were 30% heavier than equivalent rats without ORES supplementation. WT rats subjected to surgery and supplied with ORES grew more quickly, at an equivalent rate to WT rats on a normal laboratory diet. The growth of rescued KO rats supplied with ORES was significantly different than without ORES and the growth of rescued KO rats supplied with ORES was significantly different than WT surgery rats supplied with ORES (two-way ANOVA, *P*<0.01). Data show the mean±s.e.m.

Up until the time of weaning, 3 weeks after the rescue surgery, KO rats that had undergone surgery grew similarly to WT rats (Fig. 1). However, the weight curves deviated within a week of weaning, and by 7 weeks following birth (3 weeks after weaning) the KO rats were only 60% of the weight of their WT siblings. The surgically rescued KO rats also had watery fluid loss from the functional stoma, commonly had soiled fur, and appeared to be weaker. By 9 weeks after surgery, some were euthanized because of their poor condition, and only one of these rats was followed to 12 weeks. We reasoned that the KO rats, in which the rectum and most of the post-cecal colon was bypassed, may have lacked re-uptake of water and electrolyte, and they may have a deficit in the nutrition supplied by free fatty acids produced by colonic bacteria. We therefore supplied the rats with two drinking bottles, one containing regular drinking water and the other containing salts and glucose [an oral rehydration and energy solution (ORES); see Materials and Methods]. As a further test of the relevance of a possible difference between KO and WT rats, we subjected WT rat pups to the same surgery as the KO rats, and supplied these rats with the same choice of ORES or water (Fig. 1). KO rats that had undergone surgery and were supplied with ORES achieved weights 20-30% above that of rats not given ORES. They groomed themselves well and showed no signs of illness behavior. These were kept for up to 15 weeks after rescue (16 weeks of age), by which time they had reached 120-150 g in weight (Fig. 1).

WT rats that were subjected to the same surgical procedure, in which the rectum and the majority of the colon were bypassed, and were supplied with a choice of ORES and water grew at a greater rate than the equivalently treated KO rats. Interestingly, the growth curve for these WT rats after surgery and ORES was not different from the growth curve of untreated WT rats on a conventional diet (Fig. 1).

### Anatomical observations of stomas and operated colons

**Fig. 2.**
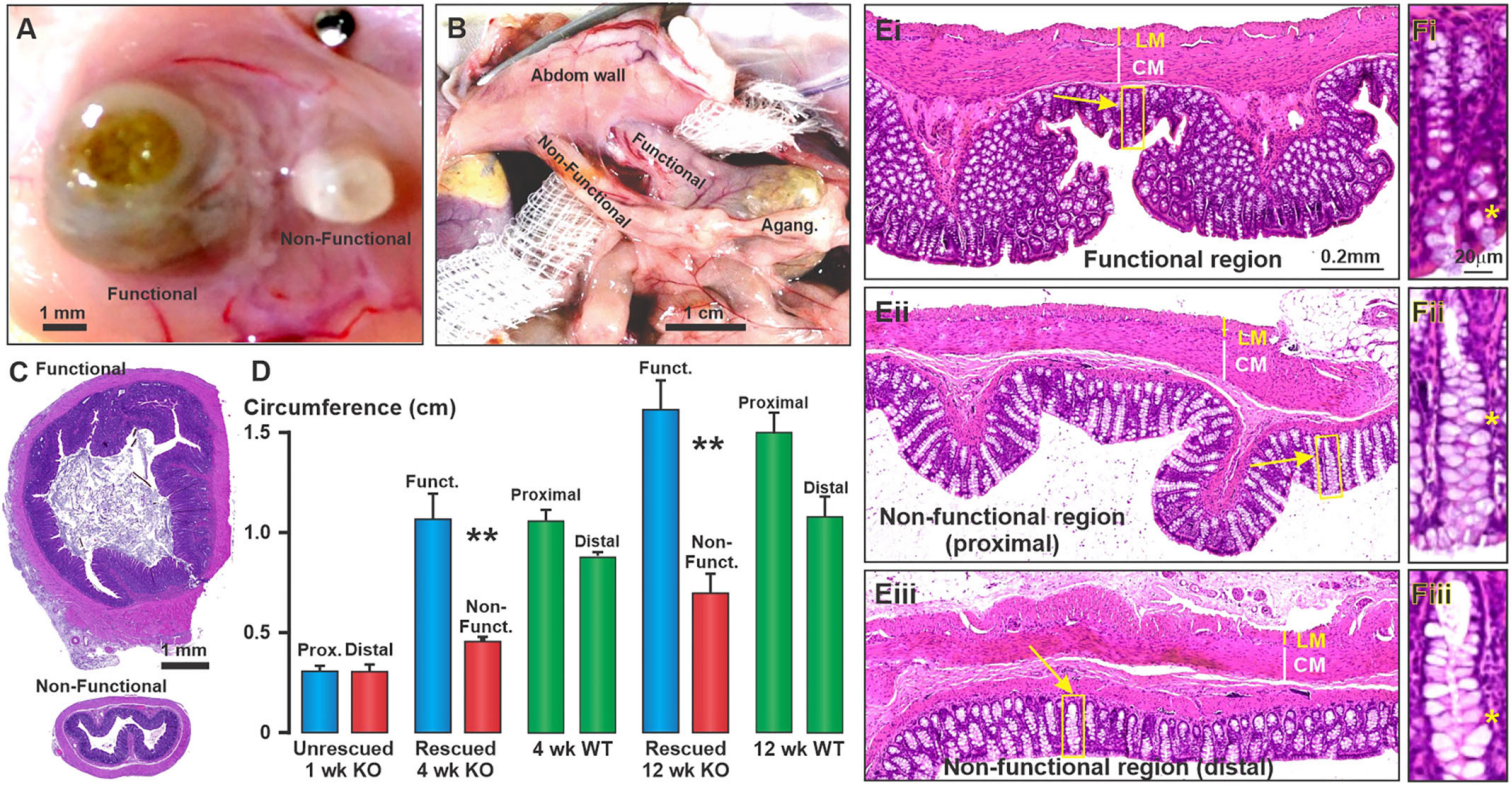
**Appearance of stomas and the colon proximal and distal to the stomas after rescue surgery.** (A-C) Stomas at 6 weeks (A) and 21 weeks (B) after surgery, and transverse sections of these regions of the colon at 21 weeks after surgery (C) in Hirschsprung (KO) rats. The images of A and B are from inside the abdominal cavity and show that the diameter of the colon at the non-functional stoma is 20-25% of that at the functional stoma, despite these having had the same diameter at the time of surgery. There is tissue continuity at the junction of the colon and the abdominal wall, and no adhesions to adjacent abdominal organs. Abdom wall, abdominal wall; Agang., aganglionic region. (D) Circumferences of the colons after they were removed at necropsy and fixed for histology. The functional colon in the KO (blue columns), proximal to the stoma, increased in circumference after creation of the stoma, but the non-functional colon (red columns) grew at a slower rate and its circumference was less than 50% of the functional colon circumference at 4 and 12 weeks of age (the circumferences of functional and non-functional colons were significantly different; **P<0.001, *n*=5). The proximal and distal circumferences were not different in the WT colons at 4 and 12 weeks of age (green columns). Data show the mean±s.e.m. (Ei-Eiii) Histological appearances of the colon from the functional and non-functional (proximal and distal) regions of a rescued rat at 12 weeks of age. The overall histology is normal. The only notable change is the greater proportions of goblet cells, in relation to other cells of the epithelial lining, in the non-functional regions; yellow arrows show mucosal glands dominated by goblet cells (Eii,Eiii). (Fi-Fiii) Enlargements of the regions in panels Ei-Eiii that are indicated by the yellow boxes (yellow arrows). It can be seen that the goblet cells (white cells, examples indicated by yellow asterisks) are more prominent in the non-functional regions. The scale bar in Ei applies to Ei-Eiii and that in Fi applies to Fi-Fiii. LM, longitudinal muscle; CM, circular muscle. Images in A-C, E and F are each representative of 12 rats.

Stomas and the connected functional colon were investigated by their anatomical and histological features for KO rats between 6 and 21 weeks after surgery (Fig. 2A-E). The colon connection to the abdominal wall was even, covered on the abdominal side by a continuous layer of parietal peritoneum, and uninflamed, and had no evidence of scarring (Fig. 2A,B). The skin repaired effectively and there was no case in which any necrotic tissue was found. Adhesions between the colon and other abdominal organs were rare and minor. No adhesions of the gastrointestinal tract to the abdominal wall away from the stoma were encountered.

The distal part of the colon that was non-functional, in that it received no content from more proximal regions, was much smaller than the functional region in KO rats by 4 weeks of age, a difference that persisted for up to 21 weeks after the surgery (Fig. 2B-D). Measurements were taken in KO rats at 4 and 12 weeks of age, at which times the non-functional colon, distal to the stomas, had less than half the circumference of the functional colon proximal to the stomas (*P*<0.001, *n*=5, two-tailed unpaired Student's *t*-test). In the WT, this difference between the proximal colon (equivalent to the functional region in the KO) and the distal colon (equivalent to the non-functional region in the KO) was not seen (Fig. 2D, green columns).

### Histological observations

The histological appearance was investigated using Hematoxylin and Eosin (H&E) staining in sections. All regions of all animals had consistently normal architecture. Although the colon diameter was reduced in the non-functional region, the muscle layers had normal thickness and there was no evidence of muscle atrophy or replacement of muscle by fibrous or connective tissue (Fig. 2Ei-Eiii). Mucosal glands in the non-functional region contained greater proportions of goblet cells and appeared to be shorter than in the same region of WT rats (Fig. 2Fi-Fiii). In KO rats at 4 weeks of age, there were 5.1±0.2 goblet cells per 50 µm of gland length (six glands from four animals), whereas in aged-matched WT rats, there were 2.8±0.2 goblet cells per 50 µm of gland length (six glands from three animals), a difference that was significant at *P*<0.0001 (unpaired two-tailed *t*-test). Gland lengths in these samples were 150.3±7.3 µm for KO and 173.9±11.0 µm for WT, which was not significantly different (*P*=0.104; unpaired two-tailed *t*-test). Large nerve bundles occurred in the distal non-functional colon in greater numbers and had greater cross-sectional areas compared with those of the proximal non-functional colon, a consistent observation across all animals from 1 week of age (Fig. S3). These bundles were at the interface between the longitudinal and circular muscle layers. Large nerve bundles at the serosal surface and crossing the longitudinal muscle were also seen. The large nerve bundles were revealed effectively using immunohistochemistry and are shown in Figs 4E-G,I,J, 5F, 6D and 7D,H).

Low-grade inflammation occurred in 12-week-old and older animals, and was most commonly seen in the mucosal lamina propria (Fig. S4). Immune cells were observed in blood vessels in the colon walls of rescued KO rats, but not in WT rats (Fig. S4).

### Patterns of innervation

We used markers of specific groups of neurons to investigate the innervation of the colon: neuronal nitric oxide synthase (nNOS or NOS1) for inhibitory motor neurons, vasoactive intestinal peptide (VIP) for intrinsic enteric neurons (both inhibitory motor neurons and secretomotor neurons), tachykinin (TK, revealed by anti-substance P, encoded by *Tac1*) for the axons of intrinsic excitatory neurons innervating the muscle, tyrosine hydroxylase (TH) for the endings of sympathetic neurons in the gut, and calcitonin gene-related peptide (CGRP, encoded by *Calca*) for sensory nerve endings emanating from spinal sensory (dorsal root ganglion) neurons.

**Fig. 3.**
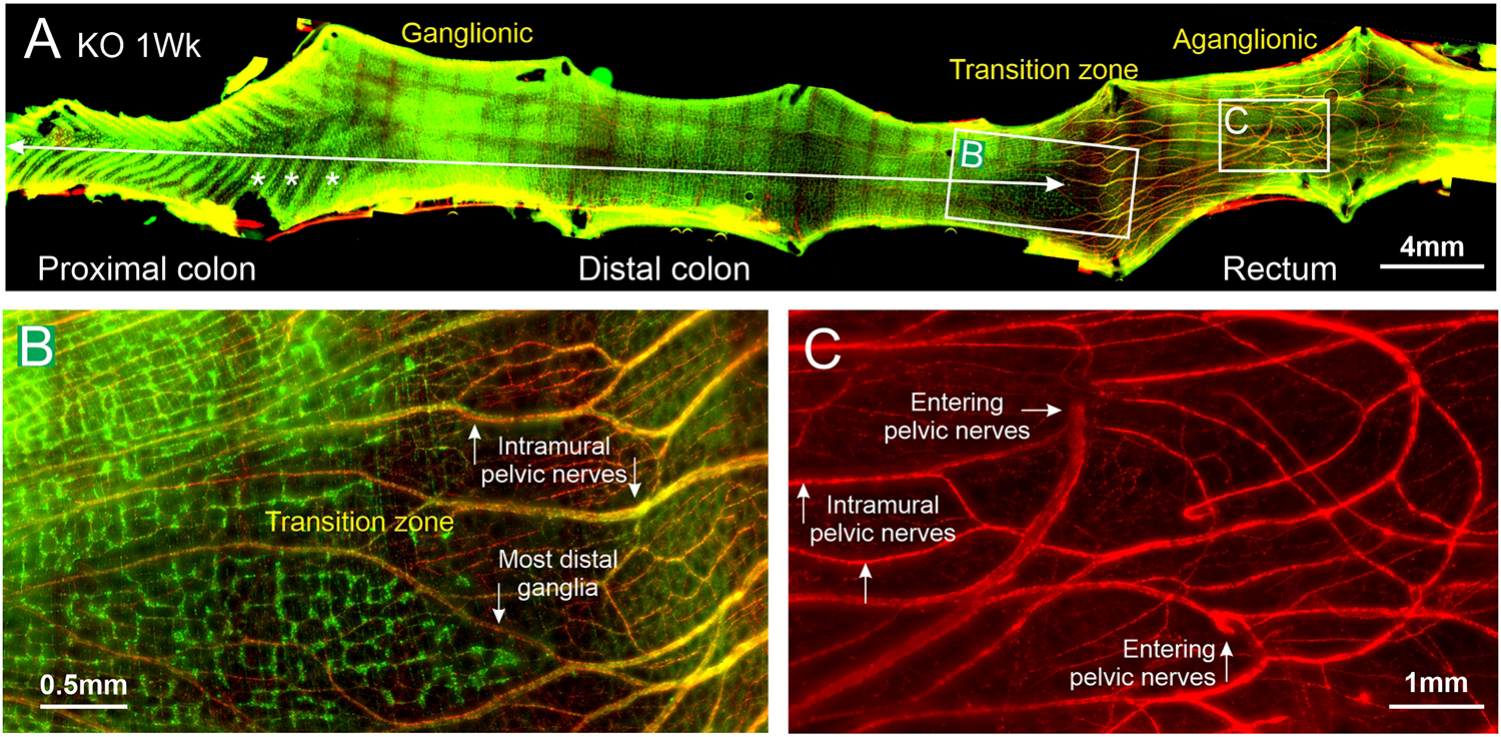
**Wholemount of the colon of a 1-week-old *Ednrb*^−/−^ rat.** (A) Low-power view, showing the mucosal folds in the proximal colon (example folds indicated by asterisks), the ganglionated plexus extending throughout almost the full length of the colon (ganglionated region marked by double-headed arrow) and the pelvic nerve branches that enter at the boundary of the distal colon and rectum (nNOS, green; TH, red). (B) The transition zone: an enlargement of the region marked B in panel A. nNOS neurons are stained in green, which reveals a ganglionated plexus to the left that ends abruptly to reveal the aganglionic region, which includes intramural branches of the pelvic nerves (arrows). Faintly in green at the right is the background autofluorescence of the mucosal glands. (C) Enlargement of the region of entry of the pelvic nerves, marked C in panel A, revealed by localization of sympathetic, noradrenergic axons using TH immunoreactivity. Branches of pelvic nerves are broken off where they enter the colorectum. Images are from an orthogonal projection of a *z*-stack and are representative of two rats.

**Fig. 4.**
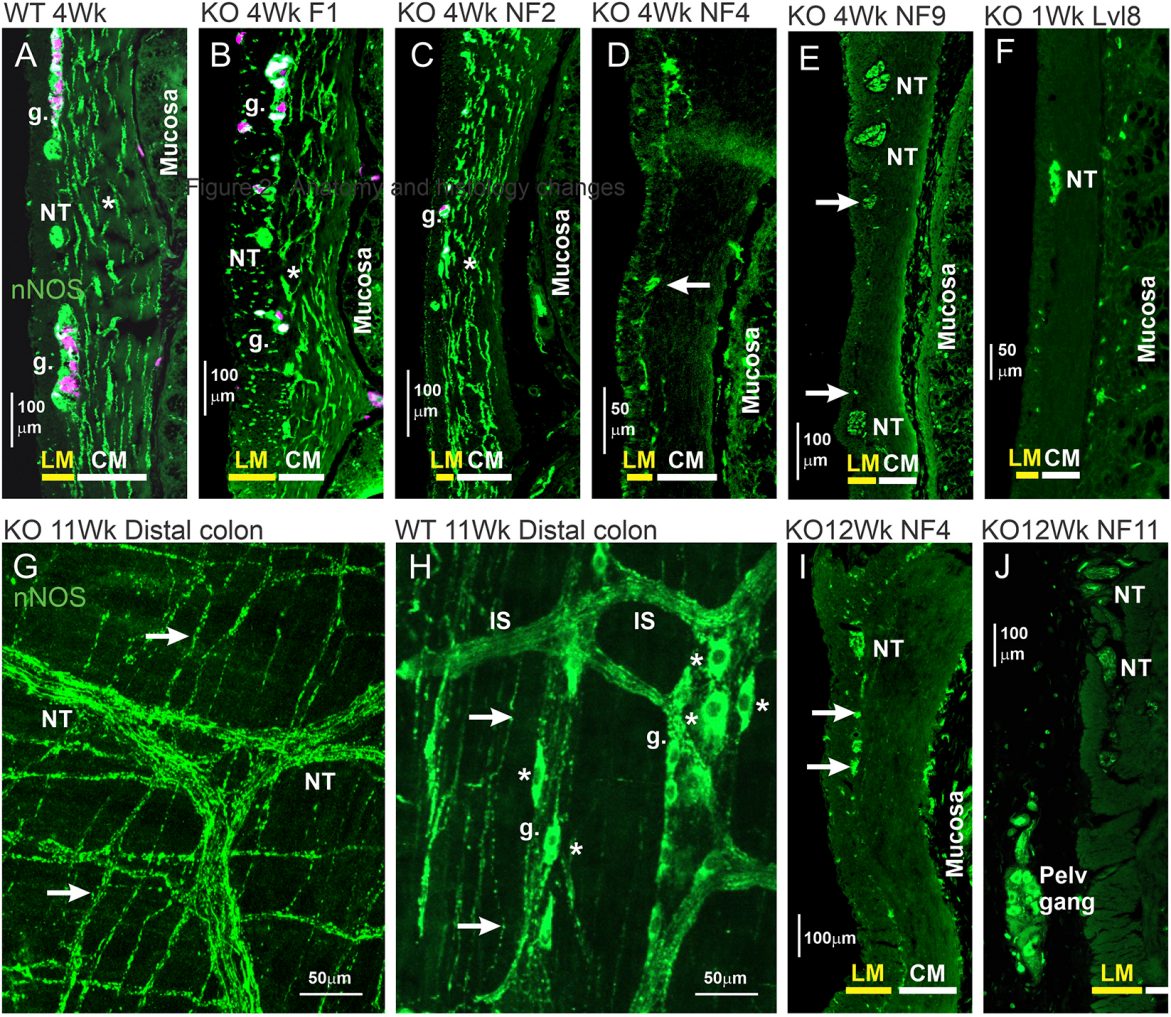
**nNOS staining in the external muscle.** nNOS staining is shown in green, staining for the Elav family members HuC and HuD indicates nerve cells in magenta; shown in transverse sections, except for panels G and H that are wholemounts. (A) Sample from a 4-week-old WT rat. There were few fibers in the longitudinal muscle (LM), whereas the circular muscle (CM) was densely innervated. (B-E) Samples from a 4-week-old *Ednrb*^−/−^ rat (3 weeks after rescue surgery). In this rat, there was a ganglionated myenteric plexus in the functional region (F1; see Fig. S1 for a diagram of regions shown here), proximal to the stoma (B). At 1 cm distal to the stoma (NF2; see Fig. S1) (C), a reduced number of enteric nerve cell bodies was found, and from 2 cm onwards (D,E), extremely few nerve cells occurred (g., ganglia). nNOS fibers (asterisks) were prominent in the CM in the control rat colon (A) and in the region proximal to the stoma (B), where they were also in increased numbers in the LM. In the hypoganglionic region distal to the stoma (C), nNOS nerve fibers were plentiful in the CM, but few innervated the LM. (F) Samples from level 18 (see Fig. S1 for levels) of a 1-week-old *Ednrb*^−/−^ rat, not subjected to rescue surgery. (G) Sample from a 11-week-old *Ednrb*^−/−^ rat. (H) Sample from a 11-week-old WT rat. (I,J) Samples from a 12-week-old *Ednrb*^−/−^ rat. nNOS fibers were rare in the muscle of the aganglionic region at all ages (D,E,F,I,J). Nerve trunks (NTs) containing nNOS fibers occurred at the level of the interface between the LM and CM in distal regions, as also seen in wholemounts (G). Smaller fiber bundles are also seen in wholemounts (arrows in G and H) and in sections (D,E,I, arrows). In WT colon (H), nerve fiber bundles (internodal strands, IS) connect ganglia (g.) that contain nNOS-immunoreactive nerve cells (asterisks). Small ganglia [pelvic ganglia (Pelv gang)] containing nNOS nerve cells were located adjacent to the rectum and the most distal part of the colon (J). Images are representative of 12 (KO) or four rats (WT).

In wholemounts, large nerve trunks entering the distal region from the pelvic plexuses were apparent (Fig. 3A-C; Fig. S5). These nerve bundles were in continuity with the intramural extensions of the pelvic nerves that have been previously described (Furness, 2006). The intramural pelvic nerves run within the colon wall at the level of the myenteric plexus, where large nerve bundles were seen in tissue sections of distal colon from the KO rats (Fig. 4F,G,I,J).

#### nNOS

nNOS is a marker of inhibitory muscle motor neurons and has been used to investigate changes in projections of motor neurons to the muscle. In transverse sections of the colon from WT rats, nNOS fibers were prominent in the circular muscle, but were few in the longitudinal muscle (Fig. 4A). Immunoreactive fibers were observed in myenteric ganglia, where nNOS positive nerve cell bodies also occurred (Fig. 4A,B). In ganglionated regions of *Ednrb*^−/−^ rats at 4 weeks of age, close to and proximal to the stomas, there was a similar innervation of the circular muscle, whereas innervation of the longitudinal muscle was substantially denser than in control regions by 4 weeks (compare Fig. 4A and 4B). Distal to the stomas, in regions with normal ganglia and in hypoganglionic regions, the innervation of the circular muscle was normal or denser than in the control colon, but there were few fibers in the longitudinal muscle (Fig. 4C), replicating the situation in the colon from WT rats (Fig. 4A). In more distal, aganglionic regions of the colon at 4 weeks, there was no nNOS innervation of the external muscle, or of other targets in the colon wall (Fig. 4D-F). This lack of innervation persisted for up to at least 12 weeks (Fig. 4I,J). However, nerve trunks containing nNOS-immunoreactive fibers were observed at the level of the interface between the longitudinal and circular muscle at all ages (Fig. 4E,F,I,J); these are deduced to be extensions of the pelvic nerves (see Discussion). Between the large nerve fiber bundles were many smaller nerve bundles, also at the level of the circular and longitudinal muscle interface, most of which were found in wholemount preparations to run in the direction of the circular muscle, but at its surface (Fig. 4G).

#### VIP

**Fig. 5.**
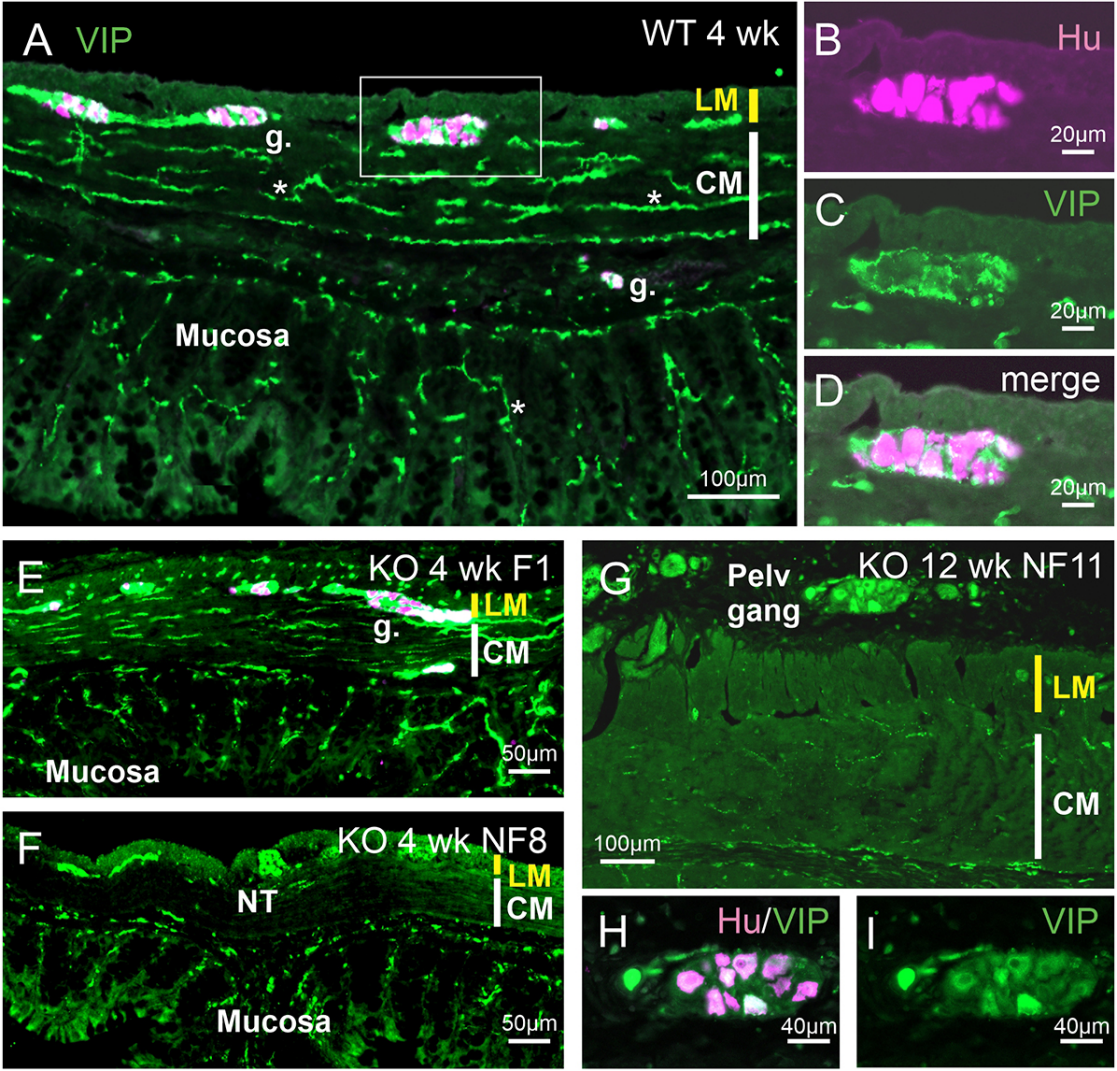
**VIP innervation of the large intestine.** VIP staining is shown in green; staining for the Elav family members HuC and HuD (Hu) to indicate nerve cells is shown in magenta. (A-D) Sample from a 4-week-old WT rat showing innervation of myenteric ganglia, one of which is enlarged in B-D, and dense innervation of the CM and mucosa (examples of fibers indicated by asterisks in A). This example is from the distal rectum. (B-D) A control (WT) myenteric ganglion showing VIP-immunoreactive fibers surrounding myenteric nerve cells. (E) The ganglionated region, proximal to the rescue stoma, from an *Ednrb*^−/−^ rat. There is conspicuous increased innervation density of the LM. (F) The aganglionic region, distal to the rescue stoma, from an *Ednrb*^−/−^ rat. Innervation of the CM is largely absent and there are nerve fiber trunks (NTs) carrying VIP fibers at the level of the myenteric plexus. (G-I) Micrographs from the rectal region of a KO rat. VIP-immunoreactive nerve cells occur in ganglia of the pelvic plexuses that are close to the rectal wall (G, enlarged in H,I), and nerve fibers are absent from the muscle layers. Examples of enteric ganglia are indicated (g.). Images are representative of 12 (KO) or four rats (WT).

VIP has been investigated as a marker of enteric inhibitory muscle motor neurons and of intrinsic secretomotor neurons. In colons of WT rats and in the ganglionated regions of the KO rats, VIP fibers provided a dense innervation of the circular muscle (Fig. 5A) and also supplied enteric ganglia (Fig. 5B-D). In the ganglionated, functional region of the colon of KO rats, there was an increased density of VIP fibers in the longitudinal muscle (Fig. 5E). However, VIP fiber innervation of the circular muscle in the aganglionic region was very sparse at all times examined (Fig. 5F,G). VIP nerve fibers, which are known to include nerve endings of enteric secretomotor neurons (Furness et al., 2014), provided a dense mucosal innervation (Fig. 5A). Similar innervation was observed in regions where the enteric ganglia had normal or close to normal density, that is, in ganglionated regions proximal to stomas (Fig. 5A) and in non-functional regions where ganglia were present. In regions where the enteric ganglia were sparse, there was also reduced innervation density in the mucosa, and where enteric neurons were absent, VIP innervation of the mucosa was substantially reduced (Fig. 5F). VIP-immunoreactive nerve cell bodies were found in the pelvic ganglia adjacent to the distal colon and rectum region (Fig. 5G-I), and in the large nerve trunks that occurred in the aganglionic regions (Fig. 5F).

#### TK

**Fig. 6.**
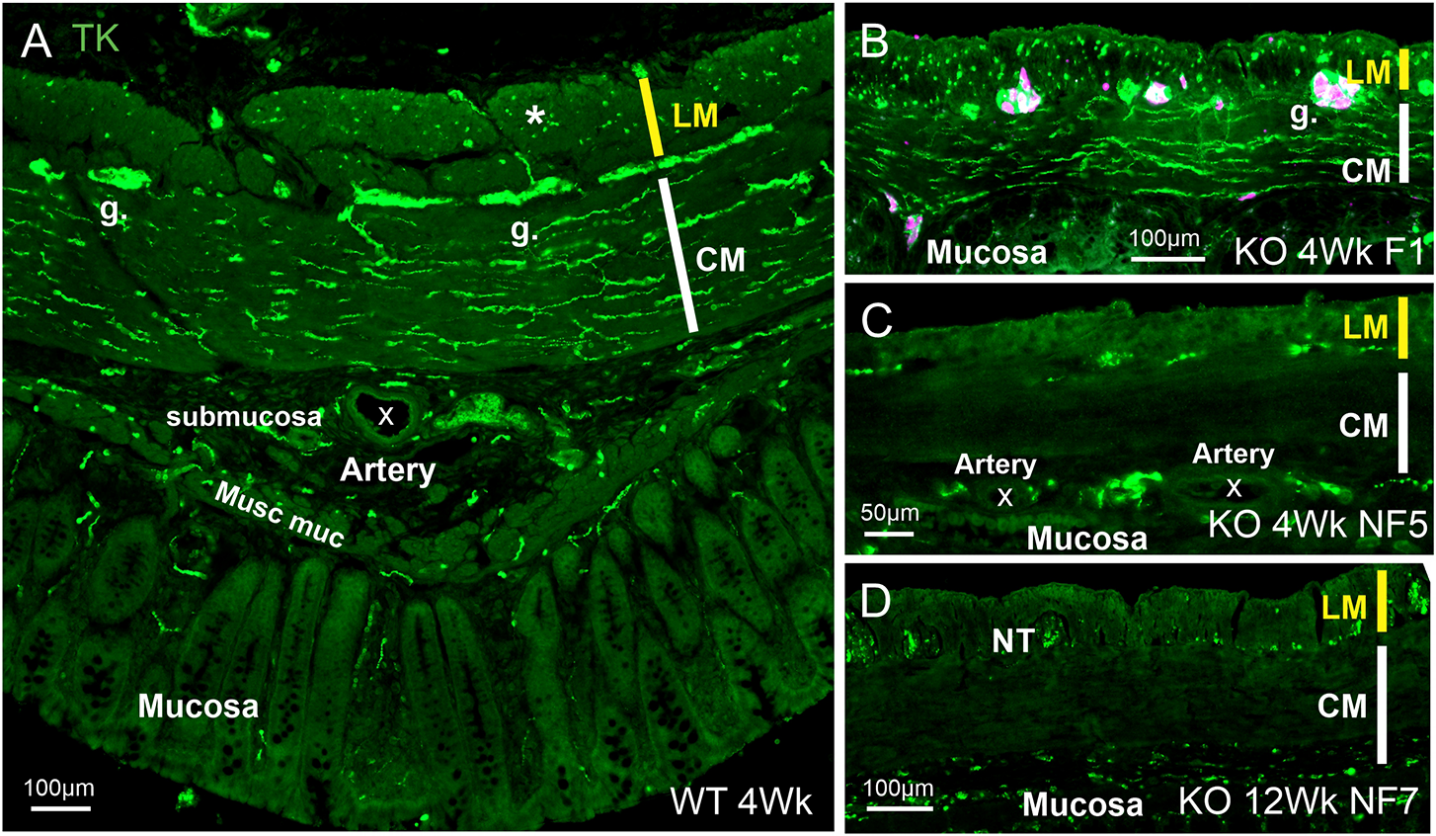
**TK innervation of the large intestine.** TK staining, revealed by an anti-substance P antibody, is shown in green; staining for the Elav family members HuC and HuD to indicate nerve cells is shown in magenta. TK immunoreactivity reveals innervation of the muscle from enteric excitatory neurons. (A) Sample from a 4-week-old WT rat showing dense innervation of the CM but sparse innervation of the LM (example at asterisk). Numerous fibers occurred in myenteric ganglia, there was innervation of arteries (marked by X in the lumen) and innervation at the base of the mucosa. Musc muc, muscularis mucosae. (B-D) Images from KO animals. (B) In the ganglionated region oral to the functional stoma, an increased density of innervation of the LM was apparent. (C,D) In the aganglionic regions, there were substantially fewer TK fibers in the CM compared to ganglionated regions of WT or KO rats, and few fibers in the LM. (C) The innervation of arteries was similar to that seen in control colon. Examples of enteric ganglia are indicated (g.). Images are representative of 12 (KO) or four rats (WT).

TK immunoreactivity is a marker of excitatory muscle motor neurons and has been used to investigate projections to the muscle. Other TK fibers innervate intramural arteries. Anti-substance P antibodies were used to localize TK-containing nerve fibers in the colon, including excitatory nerve fibers innervating the muscle. Substance P is one of a group of peptide products of the *Tac1* gene, known as tachykinins (TKs), which include neurokinin A, neuropeptide K and neuropeptide γ, and have in common a C-terminal amidated peptide region: Phe-X-Gly-Leu-Met-amide, where X is an aromatic or hydrophobic residue (Shimizu et al., 2008). In the control colon, TK-immunoreactive fibers were prominent in the circular muscle but were rare in the longitudinal muscle, except at the distal part of the rectum (Fig. 6A), where there was a denser innervation of the longitudinal muscle. There was also TK innervation of arteries in the colorectal wall and in the mesentery. TK nerve fibers were located in the submucosa and at the base of the mucosa (Fig. 6A).

In *Ednrb*^−/−^ rats, in ganglionated regions proximal to the stoma, there was a substantially increased density of innervation of the longitudinal muscle (compare Fig. 6A and 6B), whereas in more distal, aganglionic regions, the longitudinal muscle innervation was sparse or absent (Fig. 6C,D). In contrast to the ganglionic regions, and also to the colons of WT rats, there were few or no TK fibers in the circular muscle of aganglionic regions in KO rats (Fig. 6C,D). Arterial innervation in the non-functional, aganglionic region appeared normal (Fig. 6C). Immunoreactivity was observed in the nerve trunks at the longitudinal/circular muscle interface in aganglionic regions (Fig. 6D).

#### Tyrosine hydroxylase

**Fig. 7.**
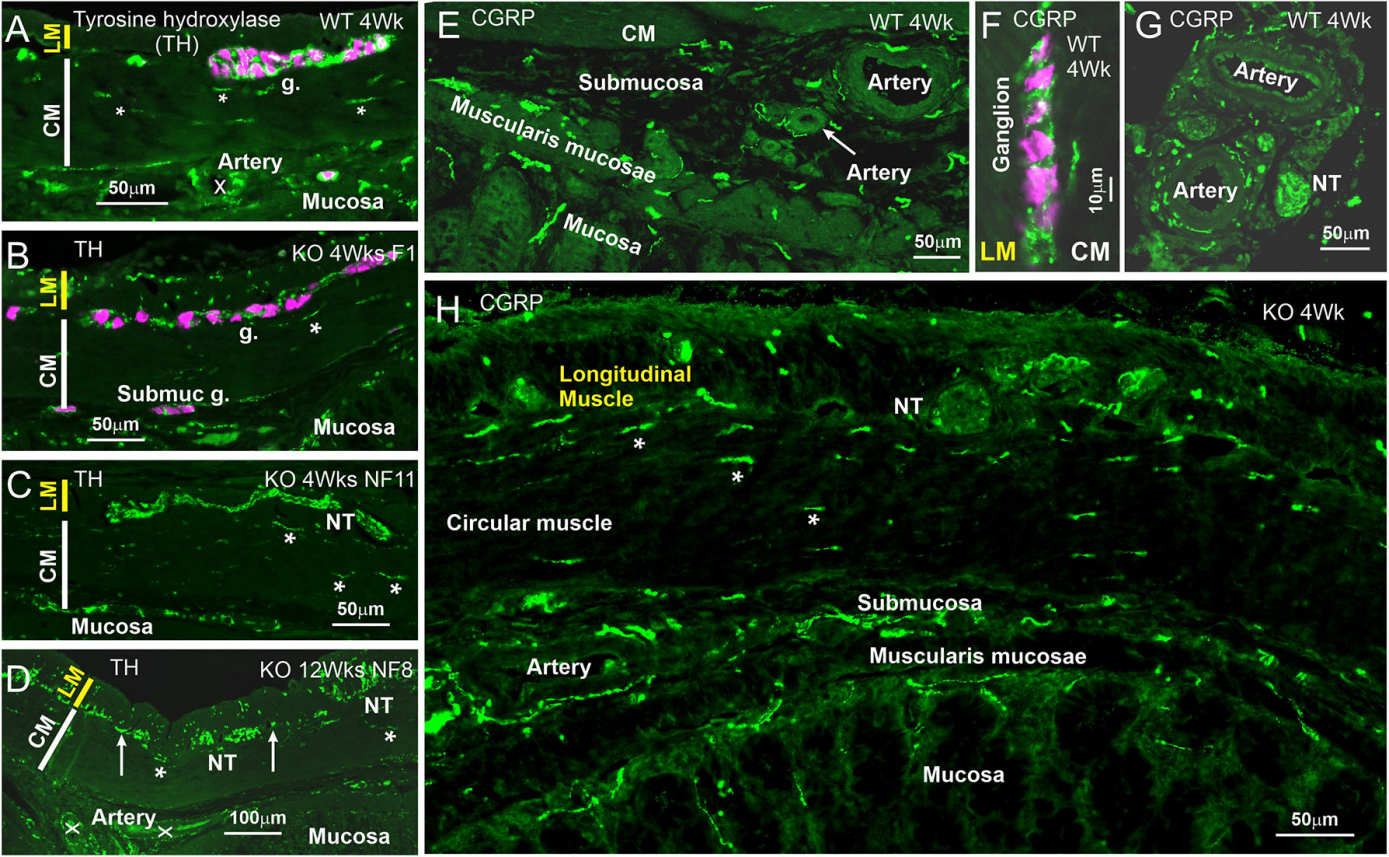
**Innervation from extrinsic sources: sympathetic and spinal afferent neurons.** (A-D) Sympathetic innervation of the large intestine [marked by tyrosine hydroxylase (TH) staining in green, and staining for the Elav family members HuC and HuD in magenta]. (A) Sample from a 4-week-old WT rat. Noradrenergic fibers innervate myenteric ganglia (g.) and intramural arteries (the arterial lumen is marked ‘X’), but are sparse in the muscle (fibers marked by asterisks) and at the base of the mucosa (arrows, also seen in panel B). (B) The colon wall from the ganglionic zone, proximal to the rescue stoma, of a 4-week-old *Ednrb*^−/−^ rat. The density of innervation of the LM is greater than that of the control, but other layers have normal innervation. (C) Section from the distal part of the colon/rectum. A large nerve trunk (NT) is seen at the interface of the LM and CM layers. Other structures are normally innervated. (D) Distal, aganglionic region, from a 12-week-old KO rat. Prominent nerve trunks (NT) are seen at the level of the myenteric plexus and small fiber bundles (arrows) are also at this level. The distributions of fibers elsewhere in the sections are normal, including around intramural arteries. (E-H) Spinal afferent innervation [marked by calcitonin gene-related peptide (CGRP) staining in green, and staining for the Elav family members HuC and HuD in magenta]. (E-G) Sections from a 4-week-old WT rat show CGRP fibers in the submucosa, at the base of the mucosa and supplying the muscularis mucosae (E), innervating neurons of myenteric ganglia (F) and supplying arteries in the colon wall (E) and around arteries in the adjacent mesentery (G). (H) The colon wall of the aganglionic region of an *Ednrb*^−/−^ rat at 12 weeks of age. The innervation is very much like that in control, except that there are no innervated ganglia and the innervation of the LM is increased. There is sparse innervation of the CM (example fibers indicated by asterisks). Images are representative of 12 (KO) or four rats (WT).

Tyrosine hydroxylase (TH), a marker of sympathetic, noradrenergic neurons, was located in nerve fibers innervating myenteric ganglia, in a small number of fibers in the circular muscle, and around small arteries, which were most commonly seen in the submucosa and at the mesenteric attachment in WT rats (Fig. 7A). There were fine nerve fiber bundles in the submucosa and a small number of fibers associated with the muscularis mucosae and the bases of the mucosal glands. At 1 week in WT rats, the arteries at the level of the submucosa had not developed, but from 4 weeks, the innervation of intramural arteries was obvious. In 4-week-old *Ednrb*^−/−^ rats, the innervation of the colon was similar to that in the WT rats, except that at the level of the myenteric plexus, there were few fibers proximally, whereas at distal sites, some large fiber bundles were seen (Fig. 7C). At 4 weeks, there was hyperinnervation of the longitudinal muscle proximal to the stoma (Fig. 7B). Also, even at 1 week in the *Ednrb*^−/−^ rats, there were large nerve trunks at the interface of the external muscle layers, most notably in the distal regions, and a sparse innervation of the circular muscle, similar to the innervation in WT. There was also innervation of the extramural and intramural arteries, comparable in density to arterial innervation in WT rats. At 12 weeks in the KO rats, the large fiber bundles were very prominent in the distal regions (Fig. 7D) and there was also, more than usual, a dense innervation of the longitudinal muscle in the distal aganglionic region.

#### CGRP

CGRP has been investigated because it is a marker of sensory axons that arise from cell bodies in the dorsal root ganglia. There are also some enteric CGRP neurons that have endings in the mucosa and enteric ganglia. In the colon from WT rats, CGRP-immunoreactive fibers were prominent in the submucosa, where they innervated intramural arteries, and at the base of the mucosa, where nerve fibers were closely associated with the muscularis mucosae (Fig. 7E-H). Fibers were sparsely arranged in the longitudinal and circular muscle and also supplied enteric ganglia. Arteries in the mesentery adjacent to the colon were surrounded by CGRP-immunoreactive fibers and there were also numerous fibers in the accompanying nerve trunks. In the *Ednrb*^−/−^ rats, which were examined between 1 and 12 weeks of age, the innervation was indistinguishable from that of the control, except for the lack of fibers supplying enteric ganglia. In particular, the sparse innervation of the circular muscle that we observed in the WT was also observed in aganglionic regions of the KO (Fig. 7H). The similarity of innervation of arteries, the mucosa and muscle in the WT and KO rats is consistent with extrinsic neurons being a major source, although some intrinsic enteric neurons of normal animals do express CGRP (Spencer et al., 2016).

### Enteroendocrine cells

**Fig. 8.**
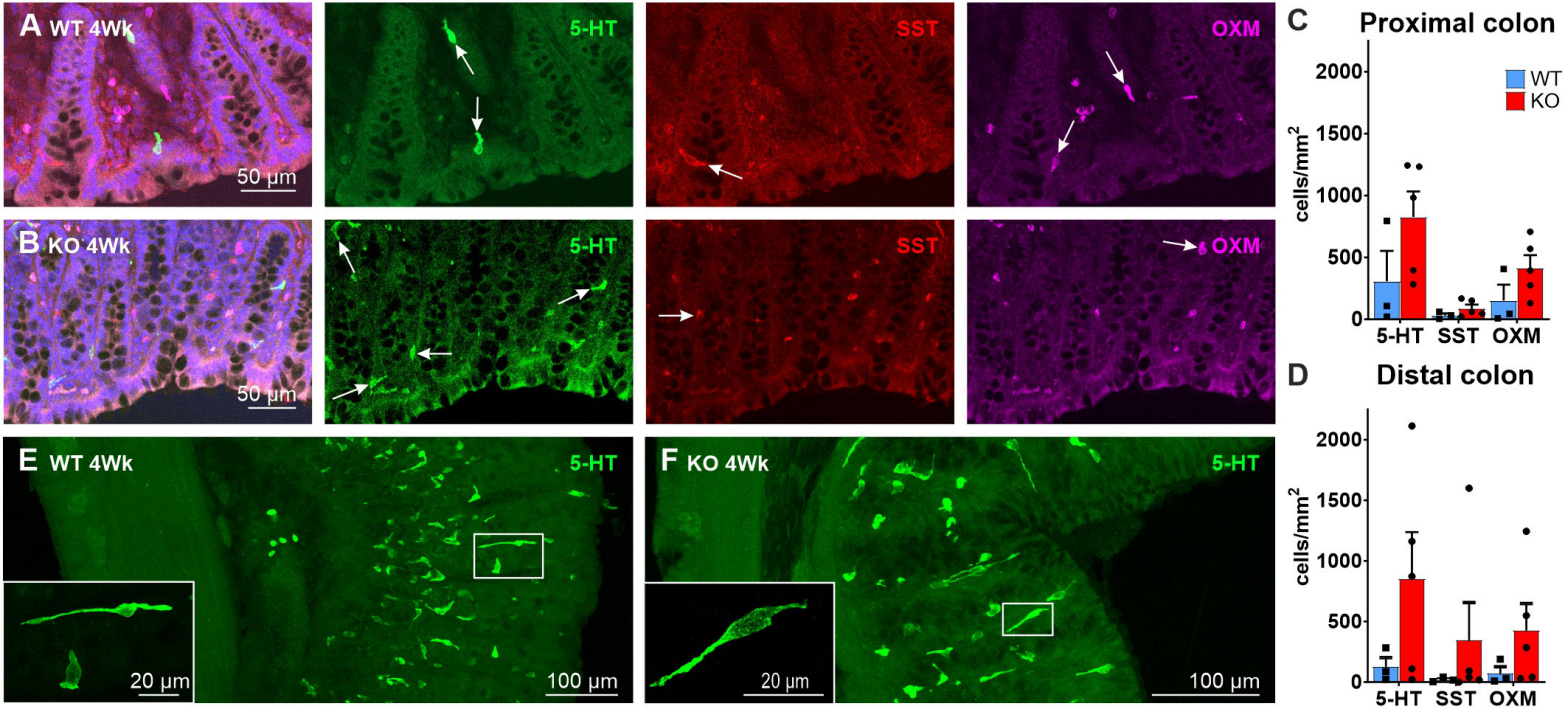
**Enteroendocrine cells in the distal colons of 4**-**week-old WT and *Ednrb*^−/−^ rats.** (A,B) Enterochromaffin (5-HT, green) cells, D-cells containing somatostatin (SST, red) and L-cells containing OXM (magenta) occurred in both WT (A) and KO rats (B). Arrows indicate EECs. (C,D) There appear to be greater numbers of EECs in KO compared to WT, but there were large variations between samples from KO rats, as indicated by dots showing the individual animal data (KO, *n*=5; WT, *n*=3). Data show the mean±s.e.m. (E,F) High-resolution (63×) inserts show examples of long basolateral processes of 5-HT cells. 5-HT cell morphology was similar between WT (E) and KO rats (F). Images are representative of four (KO) or three rats (WT).

We have investigated enteroendocrine cells (EECs) that occur in the colon: serotonin (5-HT)-secreting (enterochromaffin) cells (or simply 5-HT cells), L-cells, for which we used the marker oxyntomodulin (OXM, encoded by *Gcg*), and somatostatin (SST)-producing cells (D cells) (Fig. 8A,B). These cell types were present in all rescued KO animals and exhibited similar appearances to those in the WT rat colon. Counts of the numbers of EECs revealed large variations in the colons from the KO rats, which means that although there appeared to be greater numbers of EECs in the KO compared to WT (Fig. 8C,D), there were no statistically significant differences. Long basolateral processes of 5-HT cells were observed in thick cryosections from both WT and *Ednrb*^−/−^ rats (Fig. 8E,F).

As in our rat specimens, the types of EECs that occur in the normal human colon are also found in mucosal samples from the aganglionic region in Hirschsprung patients (Soeda et al., 1992). In humans, there were increased 5-HT, L and D cell numbers, but, as indicated, statistically significant increases in these cell types were not observed in the rat.

## DISCUSSION

### Growth of rats

Rodents, like many species, obtain energy-rich nutrition through the colonic absorption of short-chain fatty acids that are produced by colonic bacteria (Stevens and Hume, 1998), and also reabsorb water and electrolytes in the colon. To overcome potential deficiencies related to the lack of short-chain fatty acids from the colon after surgical bypass, as well as the loss of water and electrolytes, we provided the rescued rats with a drinking supply that contained both electrolytes and glucose as energy substrates. Glucose was chosen because it is more efficiently taken up in the small intestine in comparison to short-chain fatty acids. Availability of this drinking supply resulted in about 20% greater weight gain. However, when matched WT and KO (*Ednrb*^−/−^) rats were subjected to the same surgery and the same diet, the growth of *Ednrb*^−/−^ rats lagged behind their WT counterparts, having about 25% lower weight at 10-12 weeks of age. Thus, a component of the lack of growth appears to be a consequence of the genetic deficiency, rather than the surgery. This may also be the case in patients with Hirschsprung disease, in whom only 37% are above the 50% weight for age achieved in the general population, but this extrapolation from rat to human is confounded by the care given to Hirschsprung patients to ensure that their dietary needs are met (Engum and Grosfeld, 2004; Gabriela et al., 2020).

### Gross appearance and histology

When the abdomen of surgically rescued rats was opened at from 4 to 21 weeks after surgery, there were no adhesions of intra-abdominal organs and the colon appeared well vascularized and healthy. Histological investigation showed that the cellular components of all tissue layers were normal. This parallels the situation in humans, where it is difficult to recognize differences between ganglionic and aganglionic regions, or between healthy colons and colons from Hirschsprung patients, except by close examination of the ganglia and the innervation of the colon (Kapur, 2016; Yang et al., 2022). We found that the thickness of the external muscle was similar for the ganglionic and aganglionic regions, but the distal region failed to grow in diameter and a considerable size mismatch was apparent by 12 weeks after surgery. Thus, in the rat, as in humans, rejoining the colon after cell implant will require that the two regions are placed in parallel, next to each other, and a side-to-side anastomosis is created.

### Innervation

In the WT rat distal colon, there are multiple sources of innervation, including efferent innervation through the pelvic and sympathetic nerves, spinal afferent innervation and innervation from the enteric ganglia. We found that the patterns of terminals from all of these sources were altered, to differing extents, in the aganglionic colon of KO (*Ednrb*^−/−^) rats.

Comprehensive studies in humans and in experimental animals indicate that VIP and nNOS are markers of enteric inhibitory motor neurons, and TKs are markers of excitatory motor neurons that innervate the circular muscle throughout the gastrointestinal tract (Brookes et al., 2009; Furness et al., 2014; Schneider et al., 2019; Spencer and Hu, 2020). The literature indicates that the cell bodies of the motor neurons are in enteric ganglia. However, we found that innervation of the circular muscle by nNOS-, VIP- and TK-immunoreactive axons was extremely sparse in the aganglionic regions at all times investigated, up to 16 weeks, after surgical rescue. This contrasts with the nNOS, VIP and TK innervation of the circular muscle in the same region in the WT rat colon or in the ganglionated regions of the *Ednrb*^−/−^ rat. Lack of nNOS innervation of the circular muscle has also been observed in human aganglionic colon (Larsson et al., 1995). In addition, also in humans, VIP and TK innervation of the circular muscle is deficient in aganglionic regions of Hirschsprung patients (Larsson et al., 1988). Furthermore, staining for the general marker, synaptophysin, shows a substantial deficit of circular muscle innervation in the aganglionic region of *Ednrb*^−/−^ rats compared to that in normal rats (Nagahama et al., 2001). An explanation may be that the enteric motor neurons in the ganglionated regions already project to local muscle targets and do not receive stimuli, such as growth or guidance factors, that cause them to extend collaterals to the non-innervated circular muscle. By contrast with the VIP, nNOS and TK fibers, TH^+^ sympathetic, noradrenergic nerve fibers and CGRP nerve fibers, which originate from extrinsic sympathetic or dorsal root ganglia (Brookes et al., 2009; Furness et al., 2014; Schneider et al., 2019; Spencer and Hu, 2020), appear to provide a normal innervation of the circular smooth muscle of the aganglionic colon.

The findings we made of large nerve trunks at the interface of the longitudinal and circular muscle resemble observations made using cholinesterase staining and immunohistochemical localization of neurotransmitters in distal, aganglionic regions of Hirschsprung patients (Garrett et al., 1969; Larsson et al., 1988; Watanabe et al., 1999; Subramanian et al., 2017). Our observations indicate that the large nerve trunks are connected with extrinsic nerve trunks of the pelvic plexuses, a connection that has been shown directly in the lethal spotted (*Ednrb*^−/−^) mouse (Payette et al., 1987). Thus, these large nerve trunks are likely to correspond to the intramural extensions of the pelvic nerves that have been described in many species (Stach, 1971; Christensen et al., 1984; Fukai and Fukuda, 1984). It is notable that nNOS, VIP and TK fibers were found in these large trunks between the muscle layers of the aganglionic region as early as 1 week of age, and were still in this position without providing innervation within the circular muscle, up to 16 weeks of age. We conclude that these fibers may arise from pelvic ganglia but are not programmed to innervate the muscle. We observed nNOS- and VIP-immunoreactive, but not TK-immunoreactive, nerve cells in pelvic ganglia. Antibodies against TKs commonly recognize the amidated peptide products in nerve terminals, but not the precursor (TAC1 pre-pro-peptide) in the cell body, it being necessary to block transport of the precursor and products from cell bodies, e.g. with colchicine that blocks microtubular transport, in order to retain the immunoreactive products in the cell bodies (Costa et al., 1980).

We observed a substantial innervation of the longitudinal muscle by nNOS, VIP and TK in functional regions after stoma creation, although the longitudinal muscle is not innervated by such fibers in the WT. We presume that this longitudinal muscle innervation arises from the local enteric ganglia. It presumably cannot come from the large nerve trunks that are seen more distally, because these were severed when the rescue surgery was conducted to create the stomas.

In contrast to the muscle innervation, some VIP fibers innervate the mucosa in the aganglionic regions. The mucosal VIP innervation in normal animals, and in humans, arises from intrinsic secretomotor neurons with their cell bodies in submucosal ganglia, although some may arise from myenteric ganglia (Lundgren, 2002; Vanner and MacNaughton, 2004; Furness et al., 2014; Bornstein and Foong, 2018). The VIP innervation that we have observed in aganglionic regions possibly arises from neurons of the submucosal ganglia, or possibly myenteric ganglia of more proximal regions.

### General applicability

These studies were performed in an *Ednrb*^−/−^ Hirschsprung disease rat. It remains a question how this may apply in Hirschsprung disease of other genetic backgrounds. It is notable that *Ednrb* insufficiency is associated with only 3-7% of Hirschsprung disease patients (Heanue and Pachnis, 2007), whereas major characteristics that we have observed in our rat model are replicated in most Hirschsprung patients, including the large nerve trunks observed in the distal aganglionic regions that, along with aganglionosis, are regarded as diagnostic indicators of Hirschsprung disease (Subramanian et al., 2017), and the paucity of inhibitory and excitatory innervation of the circular muscle. It is unknown why there are few axons of motor neurons in the muscle of the aganglionic region. It could indicate that signals that direct these axons to their targets are deficient. As in the human disease, there were increased numbers of goblet cells and an increase in EEC numbers in the mucosa of the aganglionic region, but, overall, tissue histology was normal as has been previously observed in humans (Thiagarajah et al., 2014; Kapur, 2016; Yang et al., 2022). Thus, the *Ednrb*^−/−^ Hirschsprung disease model in rat appears to be representative of Hirschsprung disease in general. Nevertheless, animal models based on mutations of other genes require investigation to validate this conclusion.

### Conclusions

We conclude that *Ednrb*^−/−^ Hirschsprung disease rats, in which fluid and energy loss are compensated by provision of modified drinking water, survive well and exhibit many of the features of Hirschsprung disease that are observed in the colons of patients. This includes normal histology of all non-neural tissue elements in the aganglionic region, large nerve trunks in the distal region and deficiencies in the innervation of the circular muscle in the aganglionic region. Thus, the rescued *Ednrb*^−/−^ rat is predicted to provide a good model for the investigation of cell therapies for the treatment of Hirschsprung disease.

## MATERIALS AND METHODS

### Rats

Experiments were conducted on Florey sl/sl (*Ednrb*^−/−^) hooded Wistar rats. These rats were rederived from a line originally discovered by Ikadai et al. (1979) and characterized by Gariepy et al. (1996). They have a 301 bp deletion in the 3′ end of the first exon of the *Ednrb* gene. On a hooded Wistar background, *Ednrb^−/−^* progeny exhibit a characteristically changed pattern of pigmentation. *Ednrb^−/−^* (KO), *Ednrb^+/−^* (heterozygous, Het) and *Ednrb^+/+^* (WT) rats that were used in this study were bred by Het × Het matings. All experiments were approved by the Animal Ethics Committee of the Florey Institute of Neuroscience and Mental Health (Ethics Approval 19-004) and complied with the Australian Code for the Care and Use of Animals for Scientific Purposes (National Health and Medical Research Council of Australia).

### Surgery

Both KO and WT rats were subjected to surgery that, in KO rats, rescues the rats so that they do not die, but survive as long as is permitted by the research team, currently more than 5 months (Stamp et al., 2015, 2022). This is referred to as rescue surgery. Surgery was conducted on 7- to 10-day-old postnatal *Ednrb*^−/−^ (KO) rat pups weighing between 6 and 10 g. Two stomas were created, one leading the colon proximal to the aganglionic region through the skin (referred to as the functional stoma), and the other leading the distal region through the skin, the non-functional stoma (Stamp et al., 2022) (Fig. S1). From 3 weeks of age, rats were provided with ORES to facilitate weight gain and to replace fluid lost with the stool. The ORES solution consisted of 3.5 g NaCl, 1.5 g KCl, 2.9 g sodium citrate and 20 g D-glucose in 1 l of distilled water.

### Sample preparation

Tissue samples were taken from KO rat pups that had not been subjected to rescue surgery between 7 and 29 days after birth, from WT pups not subjected to surgery, and from both KO and WT rats that had been subjected to rescue surgery (Fig. S1A,B). Samples of tissue were prepared for examination in wholemounts or cryostat sections.

Colons collected from WT and KO animals at different ages were placed in PBS (0.15 M NaCl in 0.01 M sodium phosphate buffer, pH 7.2) containing nicardipine (1 µM; Sigma-Aldrich, Sydney, NSW, Australia) to facilitate muscle relaxation. The colons were opened along the mesenteric attachment and either stretched taut and pinned to balsa wood sheets mucosal side down for wholemounts, or pinned mucosal side up without stretching for sectioning. Tissues were then fixed overnight at 4°C in 2% formaldehyde and 0.2% picric acid in 0.1 M sodium phosphate buffer, pH 7.2. Preparations were cleared of fixative by three 10 min washes in dimethyl sulfoxide (DMSO), followed by three 10 min washes in PBS, and then stored at 4°C in PBS containing 0.1% sodium azide (PBS-azide).

### Immunostaining

Wholemount preparations of circular muscle, myenteric plexus and longitudinal muscle were prepared by removing the mucosa and submucosa from the fixed tissue. Preparations were blocked in NHS (10% normal horse serum in PBS with 1% Triton X-100) for 30 min at room temperature (RT) and then incubated with antibodies against markers of different classes of colon innervating neurons (Table S1) overnight at 4°C. The wholemounts were then washed (three times for 10 min) in PBS before incubation with secondary antibodies (Table S2) for 1 h at RT. Preparations were given three subsequent 10 min washes in PBS and then mounted on glass slides using mounting medium (S3023 non-fluorescent mounting medium, Dako Corporation, Carpinteria, CA, USA). An unstretched 1-week-old KO colon and a 13-day-old WT colon were stained whole without further dissection. Antibody incubations were increased to four nights at 4°C for the primary antibody and one night at RT for the secondary antibody to allow adequate antibody penetration into these samples. The entire colon was imaged as a *z*-stack tile scan on an Axioscan 7 Slide Scanner (Zeiss, Sydney, Australia).

### Histology

The unstretched, fixed colons for sectioning were first photographed to enable measurements of colon circumference, and then divided into ∼5-mm-long segments for 4- and 12-week-old animals, or ∼2.5-mm-long segments for 1-week-old animals. The following segments were taken for H&E staining (Fig. S1A, blue shaded regions) in animals that underwent rescue surgery: the segment immediately proximal to the functional stoma, the third segment distal to the non-functional stoma and the third segment proximal to the anus, or, where there was no stoma, the most distal segment of proximal colon and the third segment proximal to the anus.

All other segments were prepared for cryostat sectioning by first placing them in 30% sucrose in PBS-azide (PBS-sucrose-azide) overnight at 4°C, followed by an overnight incubation in a mixture of Optimal Cutting Temperature compound (OCT; Trajan Scientific and Medical, Ringwood, Australia) and PBS-sucrose-azide in a 1:1 ratio. Segments were then embedded in 100% OCT medium, in order of oral to anal (Fig. S1A), with the distal side of the segment oriented at the cutting surface. The tissue blocks were frozen in isopentane cooled by liquid nitrogen. Cryostat sections (10 μm; Leica CM1520 Cryostat, Leica Biosystems, Sydney, Australia) were cut and mounted onto SuperFrostPlus microscope slides (Menzel-Glaser; Thermo Fisher Scientific, Scoresby, Australia). Sections were air dried for 1 h, blocked with NHS for 30 min at RT and then incubated in diluted primary antibodies (Table S1) overnight at 4°C. Sections were then washed three times with 10 min PBS, followed by a 2 h incubation in secondary antibodies at RT. Sections were washed once with PBS and twice with distilled water for 5 min each, followed by a 5 min incubation at RT with Hoechst 33258 (10 μg/ml in distilled water; Sigma-Aldrich). Sections were then washed three times for 5 min each with distilled water, and cover-slipped with mounting medium. To locate EEC processes, cryosections of 60 μm thickness were prepared as free-floating sections. Incubations were increased to 1 h at RT for NHS blocking, three nights at 4°C for primary antibodies, overnight at RT for secondary antibodies, and 45 min at RT for Hoechst 33258. Slides were examined and imaged using an LSM800 confocal microscope (Zeiss) or an Axioscan 7 Slide Scanner (Zeiss) and processed using Zeiss Zen software. The numbers of immunoreactive EECs per mm^2^ of mucosa in sections were counted from images using ImageJ (https://imagej.nih.gov/ij/).

### Data analysis

Data are expressed as mean±standard error of the mean (s.e.m.), except where noted. *n*-values are defined where appropriate in the text; these are generally numbers of animals.

## Supplementary Material

10.1242/dmm.050055_sup1 Supplementary information
