# Supplementary material for: Development of the aganglionic colon following surgical rescue in a cell therapy model of Hirschsprung disease in rat
Source: Dis Model Mech. 2023 Apr 27;16(6):dmm050055. doi: 10.1242/dmm.050055 (PMC10163357; doi:10.1242/dmm.050055)
Supplement: Supplementary information [file dmm-16-050055-s1.pdf]

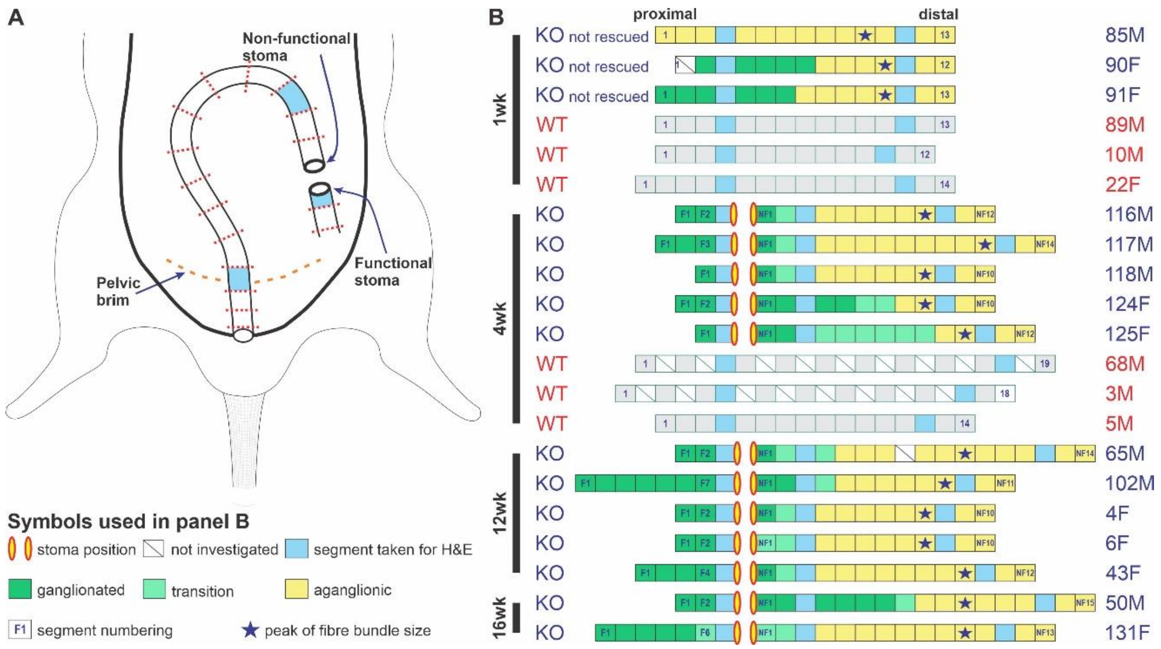

Fig. 1. Sampling sites. A: Diagram of sampling sites. Tissue samples were taken between the dotted lines, two or more samples oral to the stomas (functional colon, labeled F1 onwards) and 12-17 anal to the stomas (non-functional colon, labeled NF1 onwards), with the total numbers of samples depending on the age of the rat and the colon length. Three pieces were taken for histology (indicated in blue) and the remainder for immunohistochemistry. B: The positions of the stoma sites in relation to ganglionated, transition and aganglionic sites in colons from *Ednrb* deficient (KO) rats taken for systematic investigation in sections (animal ID and gender far right column). Sampling sites for 3 KOs taken at one week (not rescued) and wild type (WT) rats are also illustrated. Tissue from additional WT and KO rats was taken for examination in both wholemounts and sections.

Disease Models & Mechanisms • Supplementary information

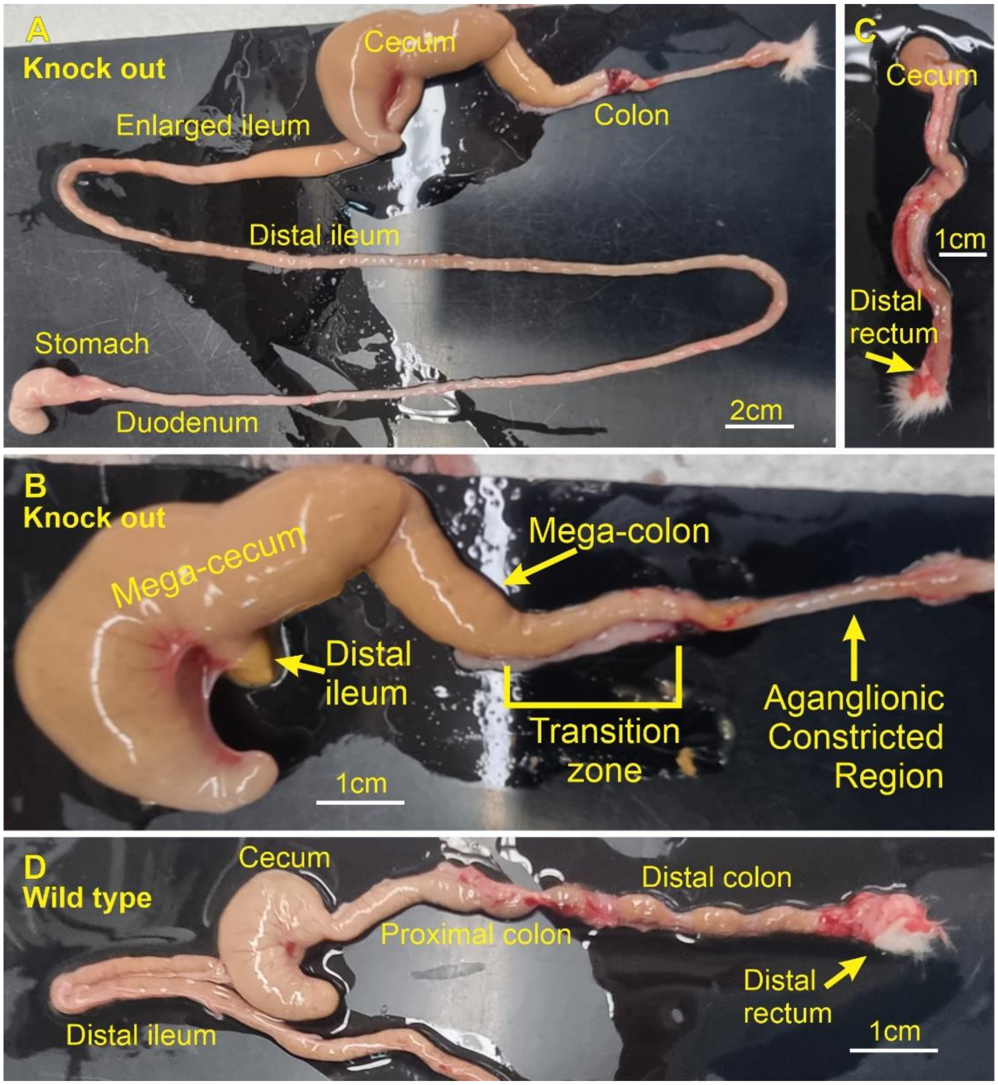

Fig. S2. Images of the freshly dissected gastrointestinal tract of an Ednrb-/- Hirschsprung rat, 28 days following birth (A, B), and the colon from another Ednrb-/- Hirschsprung rat, 24 days following birth (C), compared to the colon of a wild type rat at 28 days (D). The aganglionic distal colon region in A and B is constricted. Proximal to this was a transition zone, about 2 cm in length, where the numbers of nerve cells were fewer than normal. Further proximal is a megacolon region and an enlarged cecum. The terminal ileum is also enlarged. The colon in C is a short segment Hirschsprung case, in which almost the full length is dilated. In the wild type (D) the cecum is smaller than in the KO, the colon has a similar diameter throughout its length, and the ileum is not dilated.

Disease Models & Mechanisms • Supplementary information

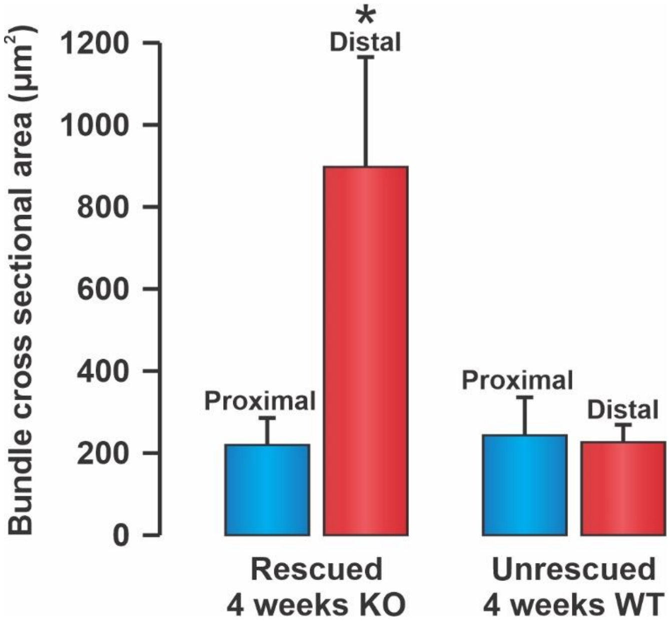

Fig. S3. Cross sectional areas of nerve bundles within the colon wall in Ednrb-/- (KO) rats and in wild type littermates at 4 weeks. The areas were significantly greater in distal regions of the KO rats, compared with proximal regions ($P < 0.05$, mean ± SD, n=4). Proximal samples were taken from the proximal end of the distal colon (WT), or the proximal end of non-functional colon (KO), 2.0-3.5 cm from the oral end. Distal samples were all 2.0-2.5 cm from the anal end.

Disease Models & Mechanisms • Supplementary information

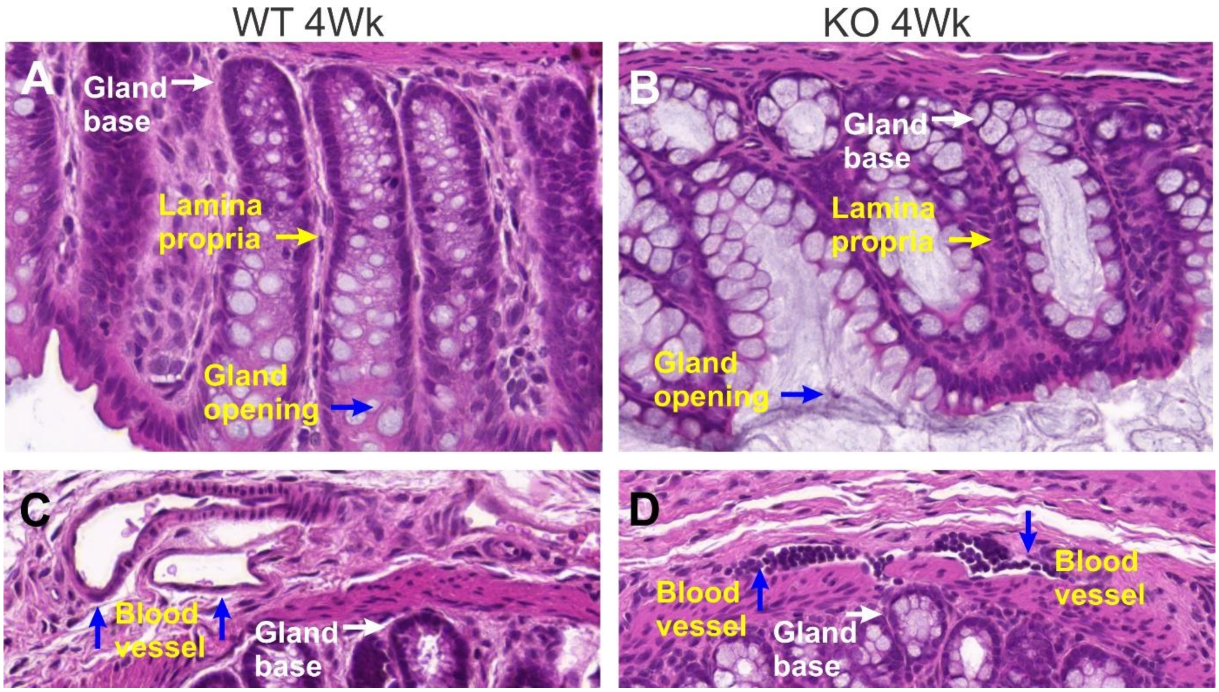

Fig. S4. Changes in colonic glands and low grade inflammation in rescued KO rats compared to WT. The mucosal appearances are compared in A and B. In the WT, goblet cells are immature at the gland base and contain only small amounts of mucin, whereas they are large and mucin-filled in the KO (indicated by arrows). The gland opening in the KO is wide and mucus filled, but narrow in the WT. In the lamina propria between the glands in WT there are mostly elongated connective tissue cells (arrow), whereas there are more numerous cells, including immune cells, in the KO. Blood vessels in the submucosa contained few or no immune cells (C), whereas occasional submucosal vessels contained many immune cells in the KO (D).

Disease Models & Mechanisms • Supplementary information

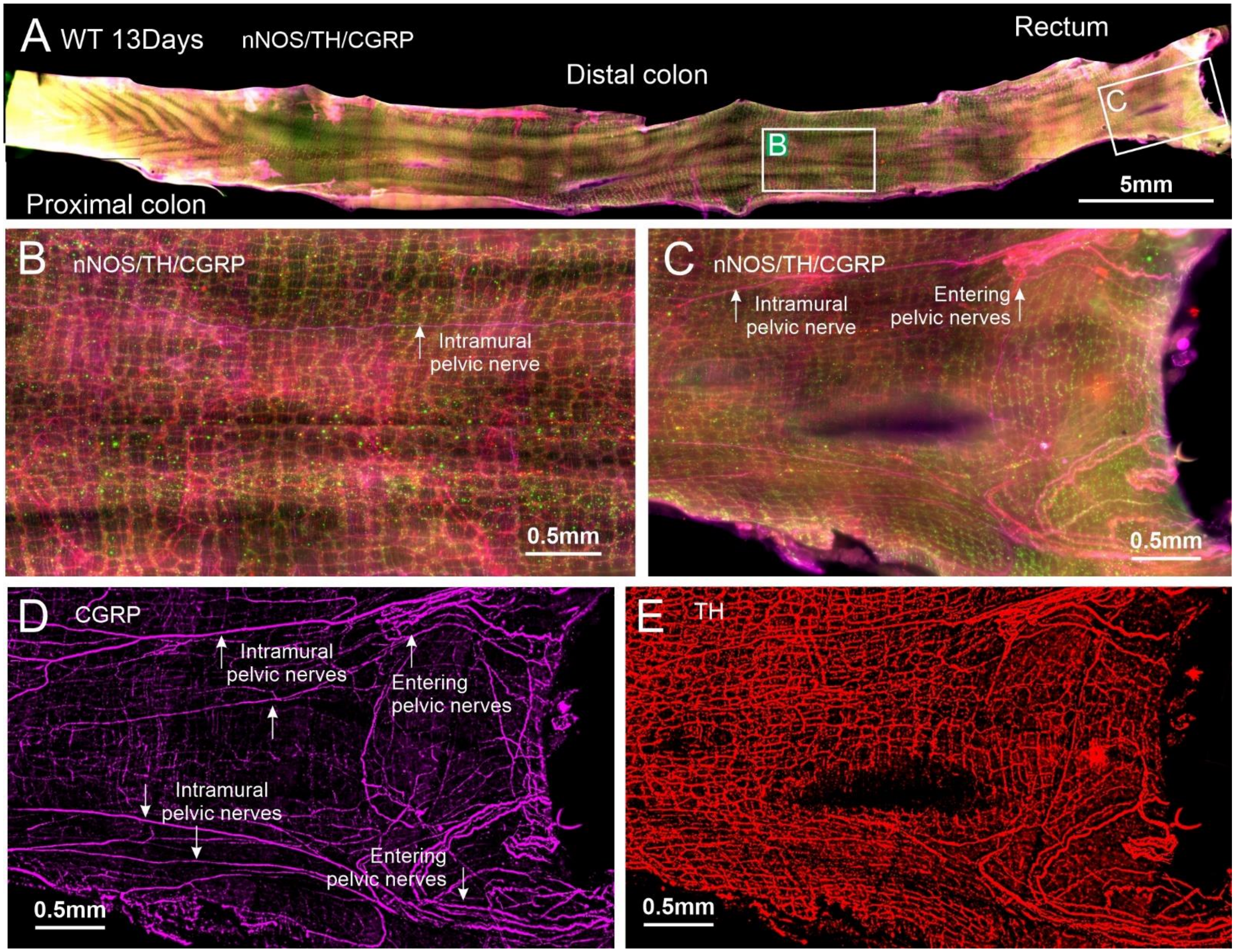

Fig. S5. Whole mount of the wild type colon, illustrating the entering pelvic nerves and the intramural pelvic nerves. The whole colon removed from a 13 day old WT rat was stained for nNOS, TH and CGRP. A: low power view, showing the triple stained colon and the regions from which the micrographs in B and C were taken. B: Distal region with normal ganglionated plexus (triple stain). Intramural pelvic nerves (arrow) tend to be obscured by the myenteric plexus and its ganglia. C: Rectum (triple stain) showing the myenteric plexus, the entering pelvic nerves and their intramural extensions (intramural pelvic nerves). D: the micrograph of C, showing only the CGRP immunoreactivity, which allows the pelvic nerves to be more clearly seen. E: the micrograph of C, showing only the TH immunoreactivity. TH fibres outline the myenteric ganglia and reveal the pelvic nerves. Images are from an orthogonal projection of a z stack.

Disease Models & Mechanisms • Supplementary information

Table S1. Primary Antibodies used

Antibody target	Host species	Dilution	Source	RRID
HuD	human	1:6000	Dr Vanda Lennon (gift)	RRID:AB_2314657
TH (tyrosine hydroxylase)	mouse	1:400	ImmunoStar Cat# 22941	RRID:AB_572268
VIP (Vasoactive intestinal peptide)	rabbit	1:400	JH Walsh (gift)	RRID:AB_2783533
Substance P	rabbit	1:800	RL Eskay (gift)	RRID:AB_2814842
HuC/HuD	Mouse, Monoclonal Antibody	1:500	Molecular Probes Cat# A-21271	RRID:AB_221448
nNOS	rabbit Monoclonal Antibody	1:200	Cell Signaling Technology Cat# 4231	RRID:AB_2152485
CGRP (Calcitonin gene-related peptide)	goat	1:1000	Arnel Products, Cat# 1780	RRID:AB_2783523
5-HT (5-hydroxytryptamine)	rabbit; polyclonal	1:1000	Immunostar Cat# 20080	RRID:AB_572263
Oxyntomodulin	mouse; monoclonal	1:2000	Ansh Labs AB-323-AO010	
Somatostatin	sheep; polyclonal	1:4000	A.A. Shulkes (gift)	

Table S2. Secondary Antibodies used

Target species/ label wavelength	Host species	Dilution	Source	RRID
Mouse 647	donkey	1:500 - 1:1000	Thermo Fisher Scientific Cat# A-31571	RRID:AB_162542
Human 647	goat	1:400	Thermo Fisher Scientific Cat# A-21445	RRID:AB_2535862
Rabbit 488	donkey	1:500 - 1:800	Thermo Fisher Scientific Cat# A-21206	RRID:AB_2535792
Goat 555	donkey	1:500	Thermo Fisher Scientific Cat# A-21432	RRID:AB_2535853
Goat 647 plus	donkey	1:500	Thermo Fisher Scientific Cat# A32849	RRID:AB_2762840
Mouse 555	donkey	1:500	Abcam Cat# ab150110	RRID:AB_2783637
